# Increased Mortality Burden in Young Asian Subjects with Dysglycemia and Comorbidities

**DOI:** 10.3390/jcm9041042

**Published:** 2020-04-07

**Authors:** Eun-Jung Rhee, Inha Jung, Hyemi Kwon, Se Eun Park, Yang-Hyun Kim, Kyung-Do Han, Yong-Gyu Park, Won-Young Lee

**Affiliations:** 1 Department of Endocrinology and Metabolism, Kangbuk Samsung Hospital, Sungkyunkwan University School of Medicine, 29 Saemunan-ro, Jongno-gu, Seoul 03181, Korea; hongsiri@hanmail.net (E.-J.R.); ina.jung@samsung.com (I.J.); hyemi.kwon@samsung.com (H.K.); seraph.park@samsung.com (S.E.P.); 2 Department of Family Medicine, Korea University Hospital, College of Medicine, Korea University, Seoul 02841, Korea; mrchir00@gmail.com; 3 Department of Statistics and Actuarial Science, Soongsil University, 369 Sangdo-ro, Dongjak-gu, Seoul 06978, Korea; hkd917@naver.com; 4 Department of Medical Statistics, Biomedicine & Health Sciences, The Catholic University College of Medicine, 222 Banpo-daero, Seocho-gu, Seoul 06591, Korea

**Keywords:** prediabetes, diabetes mellitus, mortality, comorbidity, Korean National Health Insurance Corporation

## Abstract

Background: High blood glucose level has a linear relationship with all-cause mortality. However, the influence of glycemic abnormality on mortality differs by age group. We aimed to analyze all-cause mortality according to glycemic status, age groups, and comorbidities using a national health database. Methods: The 6,330,369 participants who underwent Korean National Health Screening in 2009 were followed up until 2016, with a median follow-up of 7.3 years. All-cause mortality rates were analyzed according to glycemic status (normoglycemia, impaired fasting glucose [IFG], newly diagnosed diabetes, diabetes duration <5 years, diabetes duration ≥5 years), age groups (20–39, 40–65, and ≥65 years), and comorbidities using the Korean National Health Insurance System database. Results: At baseline, 712,901 (11.3%) subjects had diabetes. Compared with subjects without diabetes, those with diabetes at baseline showed increased mortality risk after adjustment for multiple risk factors (hazard ratio [HR] 1.613; 95% confidence interval [CI] 1.598,1.629), and those with IFG showed a significantly increased mortality risk compared with normoglycemic subjects (HR 1.053; 95% CI 1.042,1.064). Mortality risk associated with glycemic status decreased gradually from younger to older age groups and was consistently higher in those with diabetes with coronary heart disease, ischemic stroke or decreased renal function than those without comorbidities. Conclusion: Compared with normoglycemic subjects, subjects with diabetes and IFG had an increased mortality risk and the mortality risk was higher in the younger age group than in the older age group. The presence of diabetes and comorbid diseases synergistically increased mortality risk.

## 1. Introduction

The prevalence of diabetes is increasing rapidly worldwide, especially in the Asia-Pacific region [1,2,3]. Although the life-expectancy of patients with diabetes has been prolonged, the increasing incidence of diabetes in young adults is a new serious trend worldwide and especially in the Asia-Pacific area [4]. The increased prevalence of young-onset diabetes has raised concerns because longer years of life were lost to diabetes, subsequently resulting in a poor quality of life. In addition, diabetes has been highly ranked as a cause of death globally over the last decade [5]. Since diabetes is a complex disease with multiple complications, the actual mortality attributable to diabetes could be higher. 

Prediabetes is a condition that precedes diabetes and is defined by blood glucose levels higher than normoglycemia but not reaching the diabetic status [6]. Although diabetes and prediabetes are separate conditions that are defined by definite cutoff levels of blood glucose, these two conditions are considered as a continuum. Compared with normoglycemic subjects, subjects with the prediabetic status have an increased risk for cardiovascular and chronic kidney diseases [7,8,9]. In addition, mortality rate increases as the glycemic status deteriorates from normoglycemia to diabetes [10].

Diabetes causes multiple complications that increase the mortality rate [11,12]. In a recent study that included 271,174 patients with diabetes registered in the Swedish National Diabetes Register, who were matched with 1,355,870 controls, the relative importance of risk factors for predicting death and other cardiovascular diseases was significantly different, suggesting different effects of coexisting conditions on the outcome of these patients [13]. In addition, among various diabetic complications, the coexistence of which complications would shorten life expectancy more was not clearly defined.

Although the presence of diabetes affects life expectancy, the outcome differs according to age at the onset of diabetes. In a study comprising 318,083 patients with diabetes, who were matched with under 1∙6 million controls and followed up for 5.6 years, patients who were diagnosed at younger ages showed significantly increased risks for most serious outcomes compared with the controls [14]. Interestingly, if the patient was diagnosed with diabetes at >80 years of age, the hazard ratio for the outcomes was <1, suggesting that by this age, survival in those with diabetes was the same as that in the controls. However, similar analyses were not performed in Asians.

In this study, using the Korean National Health Insurance System (NHIS) database, 6,330,369 participants who underwent Korean National Health Screening (KNHS) in 2009 were followed up until 2016, and all-cause mortality rates were analyzed according to baseline glycemic status. In addition, all-cause mortality risk was specifically analyzed according to baseline risk factors, comorbidities, and age groups.

## 2. Methods

### 2.1. NHIS Database and NHIS Health Checkup Data

Nearly all Koreans (97.2% of the Korean population, approximately 50 million) are covered by the Korean NHIS, which is a nonprofit, single-payer organization by the Korean government. The NHIS maintains patients’ demographic information, examinations data, claims for disease diagnosis codes of the International Classification of Diseases (ICD-10), and treatment records that can be used to produce a population-based cohort [15]. Insured Korean adults aged >40 years and employees aged >20 years undergo regular health checkups provided by the NHIS every 1 or 2 years. 

There were two types of NHIS databases available to the researchers: one was a customized research database, composed of data from all Koreans, and the other was a sample research database, composed of data from a select group of one million Koreans. This study was performed using the customized research database that included all data available regarding our customized variables and follow-up years. Our analyses were performed after NHIS approval for the use of its database for this research (NHIS-2019-1-248). 

Our study protocol was approved by the Institutional Review Board of the Kangbuk Samsung Hospital (KBSMC2019-01-034). The requirement for informed consent was waived as the data released to the researchers were de-identified.

### 2.2. Measurements 

Body weight (kg) and height (cm) were measured using an electronic scale, and waist circumference (WC; cm) was measured at the middle point between the rib cage and iliac crest by trained examiners. All blood samples were collected after fasting, and blood pressure was measured using a sphygmomanometer after 5 min of rest. Baseline health behaviors such as income, smoking, alcohol consumption, and exercise were confirmed using standardized questionnaires. Body mass index (BMI) was calculated as body weight (kg) divided by height (m) squared.

Participants were divided into three groups according to their smoking status (never smokers, ex-smokers, and current smokers) and into three groups according to their alcohol consumption status (non-drinkers, drinking <30 g/day [moderate drinker], and drinking ≥30 g/day [heavy drinker]). Physical activity was defined as engaging in regular exercise (either of the following intensity levels): physical activity with high intensity for >20 min per session ≥3 days per week and physical activity with moderate intensity for >30 min per session ≥5 days per week.

### 2.3. Study Design and Definition of Diseases

All participants included in KNHS from January 2009 to December 2009 (*n =* 10,505,818) were initially enrolled in the study. Participants younger than 20 years (*n =* 15,327) and those with missing data for baseline characteristics and covariates (*n =* 4,160,122) were subsequently excluded, and the number of final study population was 6,330,369.

The mortality status at the end of 2016 was assessed as the primary outcome. The risk of all-cause mortality was analyzed in groups categorized by baseline glycemic status assessed with respect to fasting blood glucose (FBG). Impaired fasting glucose (IFG) was defined as FBG of 100–125 mg/dL, and diabetes was defined as FBG ≥126 mg/dL or ICD-10 code E11–14 with a claim for anti-diabetic medication [6]. Newly developed diabetes was defined as FBG ≥126 mg/dL in KNHS in 2009 and no claim for ICD-10 code E11–14 or anti-diabetic medication before 2009. The duration of claims for the above codes with anti-diabetic medication prescription before 2009 was defined as the duration of diabetes. 

In addition, the risk of all-cause mortality was analyzed according to baseline risk factors, disease status, and the presence or absence of diabetes. The BMI of the participants was divided into three levels (<18.5, 18.5–25 and >25 kg/m^2^), and the renal function of the participants was divided into four groups according to the baseline estimated glomerular filtration rate (eGFR), calculated using the Modification of Diet in Renal Disease method [16]. Ischemic heart disease was defined using the claims of ICD-10 codes I21–25, and ischemic stroke was defined using code I63 or I64. The recommended cutoff values in Koreans for abdominal obesity was defined as waist circumference ≥90 cm in men and ≥85 cm in women [17]. Dyslipidemia was defined by fasting total cholesterol ≥240 mg/dL or claims of ICD-10 codes E78 and lipid-lowering medications. Hypertension was defined by blood pressure ≥ 140/90 mmHg or ICD-10 codes I10-13, I15 and medications.

### 2.4. Statistical Analysis

Comparisons of the continuous variables at baseline between participants who were alive and dead in 2016 were performed using the Student’s *t*-test. Comparisons of the categorical variables between the groups were performed using the chi-square test.

Hazard ratios (HRs) with 95% confidence intervals (CIs) were assessed using the Cox proportional hazards model by analyzing mortality risk according to baseline glycemic status, and obesity status was assessed based on BMI or abdominal obesity. We conducted multivariable adjustments for age, sex, current smoking, alcohol drinking, regular exercise, body mass index, hypertension, dyslipidemia, and chronic kidney disease, which could affect outcomes. In addition, we performed subgroup analyses using the Cox proportional hazards model with P for interaction, according to the presence or absence of the various risk factors and diseases. Mortality rate ratio (MRR) was calculated with the incidence rate of patients with diabetes divided by that of patients without diabetes.

*P* values < 0.05 were considered statistically significant. SAS version 9.3 (SAS Institute Inc., Cary, NC, USA) was used for all statistical analyses.

## 3. Results

Among 6,330,369 subjects who participated in KNHS in 2009, 712,901 (11.3%) subjects had diabetes (Table 1). The mean age of the participants was 48.1 years, and 56.4% of subjects were men. The median follow-up period was 7.3 years. Those who had diabetes at baseline were older, more obese in general and abdominally, had higher baseline FBG levels, higher blood pressure, and had a higher proportion of cardiovascular diseases than those who did not have diabetes (Table 1).

When the HR of mortality was analyzed according to baseline glycemic status, compared with subjects without diabetes, those with diabetes at baseline showed 1.6-fold increased mortality risk after adjustment for multiple risk factors (Table 2). When the mortality risk was analyzed according to baseline glycemic status, compared with normoglycemic subjects, subjects with IFG showed weak but significantly increased risk for mortality. Compared with normoglycemic subjects, subjects with newly developed diabetes showed 1.44-fold increased mortality risk, and the mortality risk gradually increased up to 1.77-fold as the duration of diabetes lengthened to ≥5 years (Table 2). Similar findings were observed when the analyses were performed separately in a different sex.

**Figure 1:**
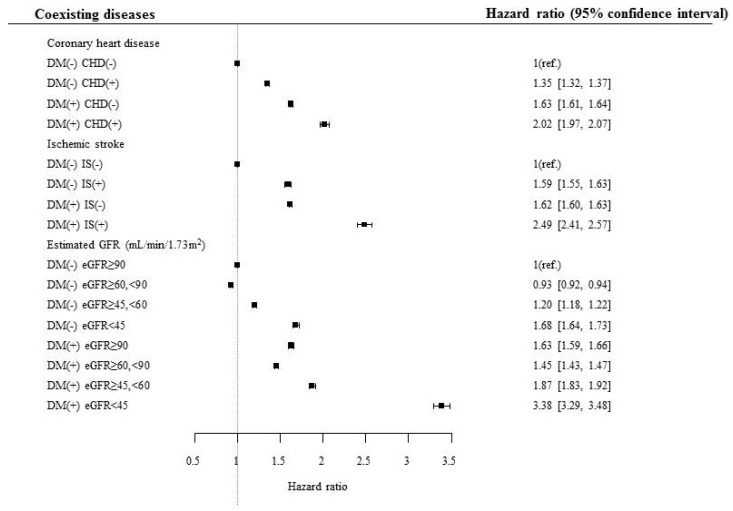
Hazard ratio for mortality according to the presence of diabetes, coronary heart disease, and ischemic stroke. DM, diabetes mellitus; CHD, coronary heart disease; IS, ischemic stroke; eGFR, estimated glomerular filtration rate.

To evaluate the effect of various diseases in association with diabetes on mortality risk, separate analyses were performed based on the presence and absence of various diseases and risk factors in association with diabetes (Table 3, Supplemental Table S1). The mortality risk was consistently higher in subjects with diabetes than in those without diabetes, although the subjects had ischemic heart disease or ischemic stroke (Table 3, Figure 1). The mortality risk gradually increased when eGFR of the subjects decreased from normal to <45 mL/min/1.73 m^2^. Subjects with diabetes and eGFR <45 mL/min/1.73 m^2^ had the highest mortality risk among the subjects divided on the basis of eGFR (3.385, 95% confidence interval [CI] 3.292–3.481), and this risk was the highest among the risks calculated for various diseases and risk factors associated with diabetes, suggesting that decreased renal function was the strongest risk factor to cause mortality in subjects with diabetes (Table 3, Figure 1).

The subjects had an increased mortality risk when they currently smoked, did not exercise, or had abdominal obesity, and these risks were higher when the subjects had diabetes than when they did not. Interestingly, compared with subjects with normal weight, those who were underweight showed significantly increased mortality risk, and this finding was also prominent in those with diabetes, with underweight diabetes subjects showing 2.3-fold increased mortality risk compared with non-diabetes, normal weight subjects (Table 3). Similar results were observed when these analyses were performed in different sexes (Supplementary Table S1).

When the above analyses were performed in different age groups, somewhat different trends were observed (Supplementary Table S2). In the age group of 20–39 years, the highest mortality risk was observed in those who had ischemic stroke and diabetes among the various diseases and risk factors (4.266, 95% CI 1.604–11.349). However, in the age group of ≥65 years, those who had eGFR <45 mL/min/1.73 m^2^ showed the highest mortality risk among the various diseases and risk factors (3.245, 95% CI 3.14–3.353), suggesting varying influence of comorbid conditions on mortality risk in subjects with diabetes in different age groups (Supplementary Table S2). 

**Figure 2:**
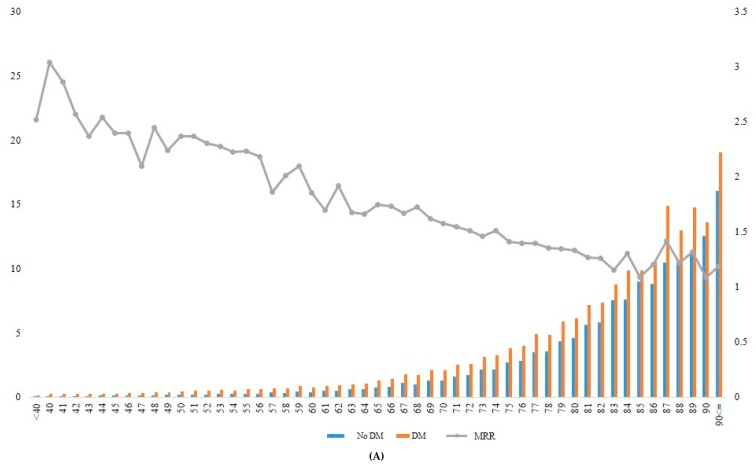

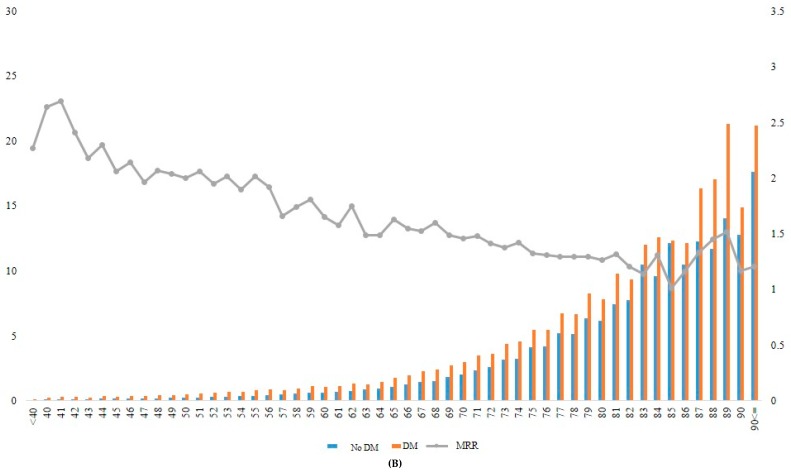

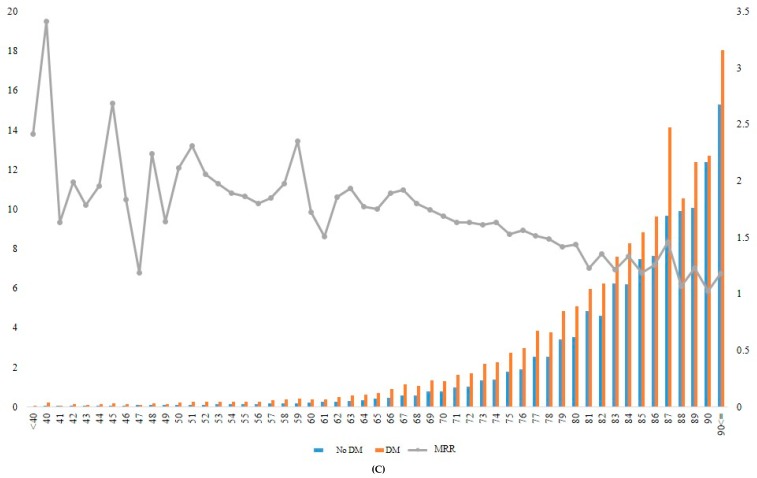
Incidence rate and MRR according to age groups in total (**A**), in men (**B**), and in women (**C**). DM, diabetes mellitus; MRR, mortality rate ratio.

When the incidence rate of mortality and MRR was analyzed according to different age groups, the MRR associated with diabetes decreased gradually from younger to older age groups (Table 4, Figure 2). For example, the patients with diabetes who were aged ≥90 years at baseline had approximately 1.2-fold increased mortality risk compared with those without diabetes in the same age group. In contrast, patients with diabetes who were aged 40 years showed 3.04-fold increased mortality compared with those without diabetes in the same age group (Figure 2A, Supplementary Table S3).

## 4. Discussion

In this large nationwide health screening and insurance database, compared with normoglycemic subjects and those with prediabetes, subjects with abnormal glucose levels had significantly increased mortality risks. Compared with non-diabetes subjects, subjects with diabetes at baseline showed 1.6-fold increased mortality risk, and the risk gradually increased as the duration of diabetes lengthened. In addition, although the presence of various diseases and metabolic risk factors increased mortality significantly, the presence of diabetes potentiated the risk across the diseases and risk factors. Among various comorbid conditions, renal dysfunction with an eGFR <45 mL/min/1.73 m^2^ showed the highest mortality risk in subjects with diabetes, and this trend was somewhat different in different age groups. When the analyses for mortality risk were performed in different age groups, mortality risk gradually decreased from younger to older age groups, suggesting a higher burden of diabetes-related mortality risk in younger subjects than in older subjects. 

Increased mortality risk in patients with diabetes has long been a great burden on the health professionals caring for them. Diabetes has been ranked as the 7^th^ leading cause of death, in the annual report of World Health Organization, although the actual rank could be higher as diabetes is a risk factor for other causes of death, such as cardiovascular disease [5]. In Korea, diabetes is the leading cause of death, and this has been observed in other Asia-Pacific regions as well [18,19]. In our study, subjects with diabetes showed 1.6-fold increased mortality risk compared with non-diabetic Korean adults after analyzing the nationwide health screening and claim data of 6,330,369 participants; the mortality risk gradually increased as the duration of diabetes increased from newly developed to <5 years to ≥5 years. 

In our study, compared with normoglycemic subjects, subjects with IFG, defined by fasting glucose between 100 and 125 mg/dL in health examinations, showed weak but significantly increased mortality risk with a HR of 1.053. Although this was a small increase, it was meaningful as the mortality risk was assessed in a large nationwide cohort. In a recent meta-analysis with 1.6 million participants, prediabetes increased the mortality risk by 1.13-fold, similar to our analysis [7]. Although the absolute excess risk was small, the fact that mortality risk actually increased in the subjects with prediabetes warrants early interventions and lifestyle modifications in these subjects. 

In the Steno-2 trial, multifactorial intervention in type 2 diabetes patients showed a 53% reduction in cardiovascular events compared with conventional intervention in type 2 diabetes patients, strongly suggesting the importance of controlling multiple risk factors for the prevention of cardiovascular diseases [20]. In the analyses from Emerging Risk Factors Collaboration, the HRs for all-cause mortality in those with diabetes were 1.9 compared with those without diabetes [21]. However, this risk increased to 3.7 or 3.8 if the subjects had diabetes and myocardial infarction or stroke simultaneously, suggesting increased mortality in patients with diabetes along with cardiometabolic multimorbidity. In addition, recent results from large cardiovascular outcome trials of novel anti-diabetic agents that control not only glucose but also multiple metabolic risk factors, reconfirms the importance of simultaneous intervention of multiple risk factors in patients with diabetes for the prevention of cardiovascular diseases [22]. Our study results showed that decreased renal function with eGFR <45 mL/min/1.73 m^2^ resulted in a 3.4-fold increased mortality risk in patients with diabetes, which is the highest among the various comorbidities. However, younger groups with ischemic stroke and diabetes showed the highest mortality risk among the comorbidities, significantly different from the older age groups, which showed the highest mortality risk in the low eGFR group, similar to the findings of the overall study. These results suggest different influences of individual comorbidities on mortality, in various age groups. 

Recent data report an increased incidence of young-onset diabetes [4]. In our study, increased mortality observed in patients with diabetes decreased gradually from younger to older age groups. For example, patients with diabetes who were aged ≥90 years at baseline had approximately 1.2-fold increased mortality risk compared with those without diabetes in the same age group. In contrast, patients with diabetes who are aged 40 years showed 3.04-fold increased mortality compared with those without diabetes in the same age group. These results suggest that there may be greater potential gains from more aggressive treatments in younger patients than in older patients with diabetes. Similar results were observed in previous studies from other ethnic groups. In a cohort study using Clinical Practice Research Datalink data from 383 general practices in England, it was found that in 187,968 patients with incident type 2 diabetes, the life expectancy differences between subjects with and without diabetes declined with age attained [23]. In the Swedish National Diabetes Registry, those who were diagnosed with diabetes at ≤40 years had 2-fold excess mortality risk relative to the controls, and this risk attenuated progressively with each increasing decade of age, similar to our study results [14]. Our results as well as previous studies suggest that the mortality risk associated with diabetes differs markedly with age at diagnosis and presumably by ethnicity, with the highest mortality risk observed in those with early diagnosed diabetes. 

In our study, compared with subjects with normal weight, those who were underweight showed significantly increased mortality risk, and this finding was also prominent in those with diabetes. Furthermore, non-diabetic obese subjects showed decreased mortality risk compared with subjects with normal body weight. In a recent study from the Taiwan Diabetes Study, a population-based retrospective cohort study, the highest hospitalization and mortality rate was observed among patients with BMI < 18.5 kg/m^2^ among groups categorized according to BMI [24]. In another study performed in sample cohorts of Korean NHIS, diabetes-related mortality risk was higher in adults with lower BMI relative to those with higher BMI, similar to the results of our study [25]. Several possible explanations exist for these results: (1) sarcopenia reflected by lower BMI in patients with diabetes is related to increased mortality [26], (2) sarcopenic obesity with reduced muscle mass and high body fat frequently associated with patients with type 2 diabetes is known be associated with increased mortality [27], (3) being underweight might mean having a lower metabolic reserve that could protect against poor prognosis or outcomes, and (4) patients with type 2 diabetes incident at a low BMI due to genetic susceptibility have worse prognosis compared with those who developed type 2 diabetes due to the metabolic stress of obesity, in whom diabetes could disappear if they lost weight [28,29]. However, the precise mechanism for increased mortality in underweight subjects is not clearly defined.

Our study has limitations. First, the definition of glycemic abnormality was evaluated only with FBG levels; HbA1c values were not available in the analyses. Therefore, there could be some inaccuracies regarding allocation of the participants. Second, definitions of the diseases were evaluated only with claim records and not with actual diagnosis. However, claim data are presumably accurate in most of the diseases. Third, since the cause of death could not be verified due to the inability to link data for cause of death with NHIS data, only the all-cause mortality rate was analyzed. Fourth, diagnosis and definition of prediabetes and diabetes could not be made due to the lack of HbA1c data in national examinations. Fifth, at least in this study, we could not include the specific data for anti-diabetic medications in the analyses. There are possibilities for biases caused by different medication usage. Despite these limitations, this study is meaningful in that, the association of diabetes, age, and comorbid diseases with mortality, were analyzed in a large nationwide database.

In conclusion, our study showed that compared with normoglycemic subjects, subjects with prediabetes had an increased mortality risk. In addition, compared with the absence of diabetes, the presence of diabetes and comorbid diseases synergistically increased mortality risk. Furthermore, the increased mortality risk was more prominent in the younger age groups than in the older age groups. Strict glycemic control and early intervention for comorbid cardiometabolic risk factors and diseases are warranted to attenuate the increased mortality risk in patients with diabetes.

## Figures and Tables

**Table 1:** General characteristics of the participants according to baseline diabetes status.

	No Diabetes (*n =* 5,617,468)	Diabetes (*n =* 712,901)	
Age (years)	46.9 ± 14.4	58.4 ± 11.7	<0.0001
Sex: men (%)	3,144,640(56.0)	427,084(59.9)	<0.0001
Body weight (kg)	64.1 ± 11.6	66.0 ± 11.4	<0.0001
Body mass index (kg/m^2^)	23.7 ± 3.2	25.1 ± 3.3	<0.0001
Waist circumference (cm)	80.2 ± 9.0	85.6 ± 8.4	<0.0001
Fasting blood glucose (mg/dL)	92.9 ± 11.4	144.3 ± 45.8	<0.0001
Total cholesterol (mg/dL)	194.9 ± 36.1	195.2 ± 41.3	<0.0001
Systolic blood pressure (mmHg)	122.5 ± 14.9	129.2 ± 15.7	<0.0001
Diastolic blood pressure (mmHg)	76.5 ± 9.9	78.9 ± 10.1	<0.0001
Obesity (BMI ≥ 25 kg/m^2^) (%)	1,809,615(32.2)	340,975(47.8)	<0.0001
Dyslipidemia (%)	1,000,705(17.8)	305,720(42.9)	<0.0001
Hypertension (%)	1,532,538(27.28)	434,475(60.94)	<0.0001
Chronic kidney disease (%)	354,747(6.3)	91,858(12.9)	<0.0001
Heart disease (%)	156,214(2.8)	41,191(5.8)	<0.0001
Ischemic stroke (%)	83,069(1.5)	18,541(2.6)	<0.0001
Current smoker (%)	1,480,548(26.4)	174,548(24.5)	<0.0001
Heavy alcohol drinker (%)	380,422(6.8)	57,953(8.1)	<0.0001
Regular exercise (%)	2,902,134(51.7)	348,223(48.9)	<0.0001

Values are expression as mean ± standard deviations or number (%).

**Table 2:** IR and multivariate-adjusted HR of mortality according to baseline glycemic status and diabetes duration.

Glycemic Status	Total Number	Number of Events	IR (Per 1000 Person Years)	Multivariate-Adjusted HR (95% CI)	
				Model 1	Model 2
Diabetes					
No	5,617,468	157,328	0.384	1.000(reference)	1000(reference)
Yes	712,901	63,298	1.244	1.547(1.533,1.561)	1.613(1.598,1.629)
Glycemic status					
No diabetes	4,176,024	103,156	0.338	1.000(reference)	1000(reference)
Impaired fasting glucose	1,427,415	52,650	0.507	1.013(1.002,1.024)	1.053(1.042,1.064)
Newly developed diabetes	186,940	10,903	0.812	1.398(1.371,1.426)	1.438(1.41,1.467)
Diabetes duration < 5 years	260,228	21,608	1.157	1.467(1.446,1.489)	1.594(1.57,1.618)
Diabetes duration ≥ 5 years	279,762	32,309	1.635	1.691(1.670,1.712)	1.774(1.752,1.797)
Men					
Diabetes					
No	3,144,640	102,064	4.459	1.000(reference)	1000(reference)
Yes	427,084	41,608	13.764	1.529(1.513,1.548)	1.605(1.585,1.623)
Glycemic status					
No diabetes	2,238,583	65,487	4.012	1.000(reference)	1000(reference)
Impaired fasting glucose	898,198	35,626	5.474	0.996(0.983,1.009)	1.044(1.031,1.058)
Newly developed diabetes	132,313	7853	8.286	1.37(1.339,1.403)	1.424(1.39,1.457)
Diabetes duration < 5 years	149,612	14,442	13.589	1.492(1.465,1.519)	1.633(1.603,1.663)
Diabetes duration ≥ 5 years	153,018	20,264	18.982	1.63(1.604,1.656)	1.731(1.703,1.759)
Women					
Diabetes					
No	2,472,828	55,264	3.049	1.000(reference)	1000(reference)
Yes	285,817	21,690	10.503	1.567(1.543,1.592)	1.629(1.603,1.656)
Glycemic status					
No diabetes	1,937,441	37,669	2.651	1.000(reference)	1000(reference)
Impaired fasting glucose	529,217	17,024	4.398	1.031(1.013,1.05)	1.061(1.042,1.081)
Newly developed diabetes	54,627	3050	7.723	1.403(1.352,1.456)	1.445(1.392,1.499)
Diabetes duration < 5 years	110,616	7166	8.897	1.404(1.369,1.44)	1.514(1.476,1.554)
Diabetes duration ≥ 5 years	126,744	12,045	13.252	1.791(1.755,1.829)	1.853(1.814,1.892)

Model 1: Adjusted for age and sex. Model 2: Adjusted for age, sex, current smoking, alcohol drinking, regular exercise, body mass index, hypertension, dyslipidemia and chronic kidney disease. IR, incidence rate; HR, hazard ratio; CI, confidence interval.

**Table 3:** The impact of various diseases and risk factors on the association of mortality risk and diabetes.

Diabetes Status	Comorbidities	Total Number	Number of Events	IR (Per 1000 Person Years)	Multivariate-Adjusted HR (95% CI)	
					Model 1	Model 2
Coronary heart disease						
No	No	5,461,254	144,092	0.361	1.000(reference)	1.000(reference)
	Yes	156,214	13,236	1.180	1.293(1.27,1.317)	1.35(1.326,1.374)
Yes	No	671,710	57,031	1.188	1.552(1.537,1.568)	1.417(1.397,1.437)
	Yes	41,191	6267	2.185	1.875(1.828,1.924)	1.737(1.69,1.787)
	Ischemic stroke					
No	No	5,534,399	149,816	0.371	1.000(reference)	1.000(reference)
	Yes	83,069	7512	1.266	1.582(1.546,1.619)	1.592(1.556,1.63)
Yes	No	694,360	59,704	1.203	1.545(1.53,1.56)	1.406(1.387,1.426)
	Yes	18,541	3594	2.847	2.402(2.324,2.483)	2.144(2.07,2.221)
Estimated glomerular filtration rate (mL/min/1.73 m^2^)						
No	>90	2,117,700	41,705	0.269	1.000(reference)	1.000(reference)
	60–90	3,145,021	89,280	0.389	0.877(0.867,0.887)	0.928(0.917,0.939)
	45–59	215,123	19,038	1.233	1.112(1.093,1.132)	1.204(1.183,1.226)
	<45	139,624	7305	0.720	1.591(1.552,1.632)	1.682(1.641,1.725)
Yes	>90	229,022	14,473	0.878	1.52(1.492,1.549)	1.432(1.402,1.463)
	60–90	392,021	32,351	1.152	1.296(1.277,1.315)	1.274(1.252,1.297)
	45–59	66,923	10,624	2.278	1.649(1.614,1.685)	1.622(1.583,1.663)
	<45	24,935	5850	3.523	3.001(2.919,3.085)	2.886(2.799,2.975)
Smoking						
No	No	4,136,920	115,403	0.382	1.000(reference)	1.000(reference)
	Current	1,480,548	41,925	0.389	1.672(1.652,1.692)	1.545(1.526,1.564)
Yes	No	538,353	47,401	1.231	1.56(1.543,1.577)	1.402(1.381,1.423)
	Current	174,548	15,897	1.286	2.522(2.479,2.565)	2.157(2.115,2.2)
Regular exercise						
No	No	2,715,334	99,493	0.504	1.000(reference)	1.000(reference)
	Yes	2,902,134	57,835	0.272	0.756(0.749,0.764)	0.786(0.777,0.794)
Yes	No	364,678	40,383	1.565	1.571(1.553,1.59)	1.42(1.398,1.442)
	Yes	348,223	22,915	0.914	1.154(1.137,1.171)	1.073(1.054,1.093)
Abdominal obesity (Men: 90 cm/Women: 85 cm)						
No	No	4,121,743	105,470	0.351	1.000(reference)	1.000(reference)
	Yes	1,495,725	51,858	0.475	0.862(0.853,0.871)	1.197(1.182,1.213)
Yes	No	364,402	34,230	1.323	1.591(1.571,1.61)	1.42(1.397,1.443)
	Yes	348,499	29,068	1.162	1.349(1.331,1.366)	1.619(1.589,1.649)
Body mass index (kg/m^2^)						
No	<18.5	2,406,712	77,986	0.445	1.446(1.428,1.464)	1.412(1.395–1.430)
	18.5–25	1,401,141	36,030	0.352	1.000(reference)	1.000(reference)
	25-	1,809,615	43,312	0.327	0.965(0.952,0.979)	0.957(0.943–0.970)
Yes	<18.5	188,227	25,411	1.934	2.443(2.404,2.483)	2.030(1.991–2.069)
	18.5–24.9	183,699	15,523	1.180	1.63(1.6,1.661)	1.375(1.345–1.405)
	≥25-	340,975	22,364	0.909	1.495(1.47,1.52)	1.262(1.237–1.286)

Model 1: Adjusted for age and sex. Model 2: Adjusted for age, sex, current smoking, alcohol drinking, regular exercise, body mass index, hypertension, dyslipidemia, chronic kidney disease and diabetes duration. IR, incidence rate, HR, hazard ratio; CI, confidence interval.

**Table 4:** IR and multivariate-adjusted HR of mortality according to baseline glycemic status in different age groups.

Glycemic Status	Total Number	Number of Events	IR (Per 1000 Person Years)	Multivariate-Adjusted HR (95% CI)	
				Model 1	Model 2
20–39 years					
Diabetes					
No	1,877,765	6589	0.478	1.000(reference)	1.000(reference)
Yes	42,780	375	1.204	2.066(1.859,2.294)	1.845(1.658,2.053)
Glycemic status					
No diabetes	1,563,198	5296	0.461	1.000(reference)	1.000(reference)
Impaired fasting glucose	314,256	1291	0.562	1.063(1,1.13)	1.03(0.968,1.096)
Newly developed diabetes	29,237	232	1.089	1.923(1.685,2.194)	1.735(1.518,1.983)
Diabetes duration < 5 years	10,356	106	1.405	2.375(1.958,2.881)	2.062(1.693,2.511)
Diabetes duration ≥ 5 years	3498	39	1.538	2.596(1.894,3.56)	2.249(1.637,3.088)
40–64 years					
Diabetes					
No	2,988,010	49,782	2.273	1.000(reference)	1.000(reference)
Yes	434,801	18,641	5.915	1.838(1.808,1.869)	1.845(1.812,1.88)
Glycemic status					
No diabetes	2,117,487	32,457	2.089	1.000(reference)	1.000(reference)
Impaired fasting glucose	862,911	17,010	2.696	1.063(1.044,1.084)	1.09(1.069,1.11)
Newly developed diabetes	121,376	3963	4.507	1.589(1.537,1.643)	1.589(1.537,1.643)
Diabetes duration < 5 years	164,923	6875	5.730	1.853(1.805,1.902)	1.931(1.879,1.983)
Diabetes duration ≥ 5 years	156,114	8118	7.197	2.1(2.049,2.153)	2.104(2.051,2.158)
≥65 years					
Diabetes					
No	751,693	100,957	18.929	1.000(reference)	1.000(reference)
Yes	235,320	44,282	27.251	1.488(1.471,1.504)	1.553(1.536,1.57)
Glycemic status					
No diabetes	495,339	65,403	18.583	1.000(reference)	1.000(reference)
Impaired fasting glucose	250,248	34,349	19.381	1.004(0.991,1.017)	1.043(1.029,1.057)
Newly developed diabetes	36,327	6708	26.800	1.314(1.281,1.347)	1.357(1.323,1.391)
Diabetes duration < 5 years	84,949	14,627	24.668	1.382(1.357,1.407)	1.499(1.472,1.527)
Diabetes duration ≥ 5 years	120,150	24,152	29.333	1.635(1.611,1.659)	1.716(1.69,1.742)

Model 1: Adjusted for age and sex. Model 2: Adjusted for age, sex, current smoking, alcohol drinking, regular exercise, body mass index, hypertension, dyslipidemia and chronic kidney disease. IR, incidence rate; HR, hazard ratio.

## Supplementary Materials

The supplementary materials are available online at https://www.mdpi.com/2077-0383/9/4/1042/s1.
