# Supplementary material for: Increased Mortality Burden in Young Asian Subjects with Dysglycemia and Comorbidities
Source: J Clin Med. 2020 Apr 7;9(4):1042. doi: 10.3390/jcm9041042 (PMC7230603; doi:10.3390/jcm9041042)
Supplement: Supplementary file 1 [file jcm-09-01042-s001.pdf]

Supplementary Table S1. Subgroup analyses of hazard ratio for mortality according to various diseases and diabetes in different sex

Diabetes status	Comorbidities	Total number	Number of events	IR (per 1,000 person years)	Multivariate-adjusted HR (95% CI)	
					Model 1	Model 2
Men						
	Coronary heart disease					
No	No	3,055,452	94,306	4.2383	1.000(reference)	1.000(reference)
	Yes	89,188	7,758	12.1827	1.219(1.191,1.247)	1.292(1.262,1.323)
Yes	No	404,770	37,990	13.2403	1.536(1.518,1.555)	1.62(1.6,1.64)
	Yes	22,314	3,618	23.5357	1.745(1.688,1.804)	1.913(1.849,1.979)
	Ischemic stroke					
No	No	3,087,997	96,979	4.3134	1.000(reference)	1.000(reference)
	Yes	56,643	5,085	12.5795	1.534(1.491,1.578)	1.559(1.515,1.604)
Yes	No	415,545	39,282	13.3381	1.531(1.513,1.549)	1.612(1.592,1.631)
	Yes	11,539	2,326	29.856	2.231(2.141,2.325)	2.338(2.243,2.437)
	Estimated glomerular filtration rate (mL/min/1.73 m^2)					
No	>90	1,174,861	30,048	3.5102	1.000(reference)	1.000(reference)
	60-90	1,820,239	58,697	4.4274	0.84(0.828,0.852)	0.904(0.891,0.916)
	45-59	82,063	9,356	16.1385	1.046(1.022,1.071)	1.173(1.146,1.202)
	<45	67,477	3,963	8.0871	1.442(1.395,1.491)	1.561(1.51,1.614)
Yes	>90	146,712	11,165	10.6717	1.524(1.492,1.558)	1.637(1.601,1.673)
	60-90	238,650	21,727	12.8159	1.232(1.211,1.254)	1.406(1.381,1.432)
	45-59	28,916	5,494	27.8577	1.531(1.487,1.576)	1.794(1.742,1.848)

	<45	12,806	3,222	38.2464	2.717(2.62,2.819)	3.125(3.012,3.243)
	Smoking					
No	No	1,746,457	62,849	4.9442	1.000(reference)	1.000(reference)
	Current	1,398,183	39,215	3.8537	1.601(1.58,1.621)	1.467(1.448,1.487)
Yes	No	261,696	26,618	14.3721	1.528(1.506,1.55)	1.591(1.568,1.614)
	Current	165,388	14,990	12.8017	2.459(2.415,2.503)	2.39(2.347,2.434)
	Regular exercise					
No	No	1,298,424	59,762	6.3688	1.000(reference)	1.000(reference)
	Yes	1,846,216	42,302	3.1326	0.728(0.719,0.738)	0.768(0.758,0.778)
Yes	No	188,293	24,208	18.4233	1.54(1.517,1.563)	1.613(1.588,1.637)
	Yes	238,791	17,400	10.1813	1.123(1.104,1.142)	1.224(1.203,1.245)
	Abdominal obesity (Men:90cm/Women:85cm)					
No	No	2,511,793	79,066	4.3229	1.000(reference)	1.000(reference)
	Yes	632,847	22,998	5.0022	0.873(0.86,0.886)	1.295(1.273,1.317)
Yes	No	273,057	27,540	14.2822	1.581(1.56,1.603)	1.635(1.613,1.658)
	Yes	154,027	14,068	12.8507	1.316(1.292,1.339)	1.93(1.891,1.97)
	Body mass index (kg/m^2)					
NO	<18.5	1,160,951	51,731	0.61502	1.466(1.444,1.489)	1.419(1.397,1.441)
	18.5-25	850,291	24,063	0.38781	1.000(reference)	1.000(reference)
	25-	1,133,398	26,270	0.3176	0.941(0.924,0.957)	0.939(0.922,0.955)
YES	<18.5	112,986	17,515	2.25084	2.485(2.437,2.534)	2.369(2.323,2.416)
	18.5-24.9	114,228	10,553	1.30007	1.597(1.561,1.634)	1.554(1.519,1.591)

	≥25-	199,870	13,540	0.94479	1.434(1.404,1.464)	1.392(1.363,1.422)
Women						
	Coronary heart disease					
No	No	2,405,802	49,786	2.822	1.000(reference)	1.000(reference)
	Yes	67,026	5,478	11.3053	1.403(1.364,1.443)	1.417(1.378,1.458)
Yes	No	266,940	19,041	9.8554	1.57(1.544,1.596)	1.641(1.613,1.67)
	Yes	18,877	2,649	19.8986	2.067(1.987,2.149)	2.163(2.078,2.25)
	Ischemic stroke					
No	No	2,446,402	52,837	2.9457	1.000(reference)	1.000(reference)
	Yes	26,426	2,427	12.8197	1.68(1.613,1.75)	1.657(1.591,1.726)
Yes	No	278,815	20,422	10.1258	1.557(1.532,1.582)	1.621(1.595,1.649)
	Yes	7,002	1,268	26.2368	2.807(2.655,2.968)	2.84(2.686,3.004)
	Estimated glomerular filtration rate (mL/min/1.73 m^2)					
No	>90	942,839	11,657	1.684	1.000(reference)	1.000(reference)
	60-90	1,324,782	30,583	3.148	0.956(0.936,0.977)	0.983(0.962,1.004)
	45-59	133,060	9,682	10.0433	1.203(1.17,1.237)	1.249(1.215,1.285)
	<45	72,147	3,342	6.3673	1.817(1.747,1.889)	1.857(1.786,1.931)
Yes	>90	82,310	3,308	5.4957	1.44(1.386,1.497)	1.554(1.494,1.615)
	60-90	153,371	10,624	9.5509	1.431(1.393,1.47)	1.563(1.521,1.606)

	45-59	38,007	5,130	19.0645	1.815(1.755,1.877)	1.985(1.919,2.054)
	<45	12,129	2,628	32.1253	3.444(3.299,3.595)	3.775(3.615,3.943)
	Smoking					
No	No	2,390,463	52,554	2.9985	1.000(reference)	1.000(reference)
	Current	82,365	2,710	4.5209	1.976(1.901,2.054)	1.819(1.75,1.892)
Yes	No	276,657	20,783	10.3919	1.581(1.556,1.607)	1.64(1.613,1.667)
	Current	9,160	907	13.9001	2.59(2.425,2.765)	2.556(2.393,2.729)
	Regular exercise					
No	No	1,416,910	39,731	3.8328	1.000(reference)	1.000(reference)
	Yes	1,055,918	15,533	2.0016	0.816(0.801,0.832)	0.824(0.809,0.84)
Yes	No	176,385	16,175	12.7713	1.597(1.568,1.627)	1.655(1.625,1.687)
	Yes	109,432	5,515	6.9054	1.219(1.185,1.253)	1.281(1.245,1.318)
	Abdominal obesity (Men:90cm/Women:85cm)					
No	No	1,609,950	26,404	2.2383	1.000(reference)	1.000(reference)
	Yes	862,878	28,860	4.5593	0.836(0.822,0.851)	1.068(1.047,1.09)
Yes	No	91,345	6,690	10.1633	1.624(1.581,1.668)	1.641(1.597,1.686)
	Yes	194,472	15,000	10.6616	1.344(1.317,1.371)	1.722(1.682,1.763)
	Body mass index (kg/m^2)					
No	<18.5	1,245,761	26,255	0.28813	1.429(1.398,1.46)	1.417(1.386,1.448)

	18.5-25	550,850	11,967	0.29585	1.000(reference)	1.000(reference)
	25-	676,217	17,042	0.34296	0.992(0.969,1.015)	0.978(0.956,1.002)
Yes	<18.5	75,241	7,896	1.47418	2.346(2.28,2.414)	2.296(2.231,2.363)
	18.5-24.9	69,471	4,970	0.98759	1.704(1.649,1.762)	1.674(1.619,1.73)
	≥25-	141,105	8,824	0.85979	1.581(1.538,1.625)	1.526(1.484,1.569)

Supplementary Table S2. Subgroup analyses of hazard ratio for mortality according to various diseases in different age groups

Diabetes status	Comorbidities	Total number	Number of events	IR (per 1,000 person years)	Multivariate-adjusted HR (95% CI)	
					Model 1	Model 2
20-39 years						
	Coronary heart disease					
No	No	1,866,474	6,523	0.4762	1.000(reference)	1.000(reference)
	Yes	11,291	66	0.7976	1.471(1.154,1.875)	1.495(1.173,1.906)
Yes	No	42,436	370	1.197	2.063(1.856,2.293)	1.845(1.656,2.056)
	Yes	344	5	2.0104	3.24(1.348,7.787)	2.828(1.175,6.802)
	Ischemic stroke					
No	No	1,869,363	6,566	0.4786	1.000(reference)	1.000(reference)
	Yes	8,402	23	0.3724	0.648(0.43,0.976)	0.69(0.458,1.04)
Yes	No	42,588	371	1.196	2.048(1.844,2.276)	1.831(1.644,2.04)
	Yes	192	4	2.8802	4.535(1.705,12.062)	4.266(1.604,11.349)
	Estimated glomerular filtration rate (mL/min/1.73 m^2)					
No	>90	901,630	3,250	0.4923	1.000(reference)	1.000(reference)
	60-90	914,829	3,132	0.4658	0.854(0.812,0.898)	0.872(0.829,0.916)
	45-59	9,489	29	0.4151	0.877(0.608,1.265)	0.877(0.608,1.265)
	<45	51,817	178	0.4616	1.015(0.873,1.181)	1.027(0.883,1.194)
Yes	>90	20,825	196	1.2954	2.102(1.818,2.429)	1.878(1.621,2.175)
	60-90	20,611	161	1.0703	1.631(1.391,1.913)	1.489(1.268,1.75)
	45-59	374	4	1.4646	2.566(0.962,6.844)	2.26(0.847,6.031)
	<45	970	14	1.9591	3.202(1.894,5.413)	2.909(1.72,4.921)
	Smoking					
No	No	1,150,761	2,996	0.3546	1.000(reference)	1.000(reference)
	Current	727,004	3,593	0.6739	1.611(1.526,1.701)	1.58(1.496,1.669)

Yes	No	19,993	133	0.9121	2.137(1.795,2.544)	1.979(1.66,2.361)
	Current	22,787	242	1.4598	3.112(2.722,3.558)	2.81(2.453,3.22)
	Regular exercise					
No	No	826,698	2,793	0.4617	1.000(reference)	1.000(reference)
	Yes	1,051,067	3,796	0.491	0.98(0.933,1.029)	0.992(0.944,1.042)
Yes	No	17,610	169	1.3209	2.291(1.96,2.677)	2.063(1.763,2.415)
	Yes	25,170	206	1.1217	1.872(1.624,2.159)	1.684(1.457,1.946)
	Abdominal obesity (Men:90cm/Women:85cm)					
No	No	1,602,430	5,515	0.4687	1.000(reference)	1.000(reference)
	Yes	275,335	1,074	0.5331	1.041(0.975,1.112)	1.067(0.982,1.158)
Yes	No	27,127	246	1.2435	2.145(1.887,2.44)	1.936(1.701,2.204)
	Yes	15,653	129	1.1339	1.966(1.65,2.342)	1.782(1.478,2.147)
	Body mass index (kg/m^2)					
No	<18.5	126,578	358	0.3854	1.161(1.04,1.297)	1.147(1.027,1.282)
	18.5-25	1,237,961	4,182	0.46	1.000(reference)	1.000(reference)
	25-	513,226	2,049	0.5448	1.001(0.949,1.057)	0.928(0.878,0.98)
Yes	<18.5	909	20	3.0318	6.726(4.335,10.437)	6.247(4.026,9.693)
	18.5-24.9	18,096	168	1.272	2.247(1.925,2.623)	2.021(1.73,2.361)
	≥25-	23,775	187	1.0814	1.814(1.565,2.102)	1.449(1.245,1.686)
40-64 years						
	Coronary heart disease					
No	No	2,905,353	47,376	2.2248	1.000(reference)	1.000(reference)
	Yes	82,657	2,406	3.9795	1.329(1.276,1.385)	1.406(1.349,1.465)
Yes	No	416,605	17,454	5.778	1.837(1.805,1.87)	1.852(1.818,1.886)
	Yes	18,196	1,187	9.0731	2.361(2.228,2.501)	2.495(2.353,2.645)
	Ischemic stroke					

No	No	2,940,369	48,400	2.2459	1.000(reference)	1.000(reference)
	Yes	47,641	1,382	3.9609	1.441(1.366,1.52)	1.506(1.427,1.589)
Yes	No	426,444	17,961	5.809	1.829(1.798,1.861)	1.84(1.807,1.873)
	Yes	8,357	680	11.3939	2.975(2.758,3.21)	3.019(2.798,3.258)
	Estimated glomerular filtration rate (mL/min/1.73 m^2)					
No	>90	1,080,566	20,104	2.5392	1.000(reference)	1.000(reference)
	60-90	1,740,758	26,051	2.0418	0.805(0.79,0.82)	0.851(0.836,0.867)
	45-59	100,447	2,242	3.0441	0.948(0.908,0.991)	1.034(0.989,1.08)
	<45	66,239	1,385	2.8454	1.136(1.076,1.2)	1.199(1.136,1.266)
Yes	>90	170,739	7,591	6.1377	1.734(1.689,1.781)	1.757(1.711,1.805)
	60-90	228,970	8,421	5.0634	1.407(1.371,1.443)	1.503(1.464,1.543)
	45-59	23,493	1,400	8.2541	1.895(1.794,2.001)	2.045(1.935,2.16)
	<45	11,599	1,229	14.9715	4.105(3.875,4.349)	4.33(4.086,4.588)
	Smoking					
No	No	2,326,017	31,243	1.8291	1.000(reference)	1.000(reference)
	Current	661,993	18,539	3.8476	1.783(1.748,1.819)	1.686(1.653,1.72)
Yes	No	312,196	10,923	4.8037	1.828(1.788,1.869)	1.874(1.833,1.917)
	Current	122,605	7,718	8.7932	3.165(3.084,3.249)	3.041(2.962,3.123)
	Regular exercise					
No	No	1,416,716	26,034	2.5098	1.000(reference)	1.000(reference)
	Yes	1,571,294	23,748	2.0602	0.778(0.764,0.792)	0.807(0.793,0.821)
Yes	No	202,552	9,879	6.7453	1.896(1.852,1.94)	1.902(1.858,1.948)
	Yes	232,249	8,762	5.1937	1.388(1.354,1.422)	1.44(1.404,1.476)
	Abdominal obesity (Men:90cm/Women:85cm)					
No	No	2,112,334	35,367	2.2863	1.000(reference)	1.000(reference)
	Yes	875,676	14,415	2.2418	0.963(0.944,0.982)	1.293(1.263,1.323)

Yes	No	236,547	11,466	6.7139	1.947(1.906,1.989)	1.916(1.875,1.958)
	Yes	198,254	7,175	4.9695	1.645(1.603,1.688)	2.177(2.114,2.242)
	Body mass index (kg/m^2)					
No	<18.5	56,471	2,063	5.0377	2.394(2.29,2.503)	2.239(2.141,2.341)
	18.5-25	1,896,732	31,319	2.2529	1	1
	25-	1,034,808	16,400	2.1612	0.864(0.848,0.88)	0.86(0.843,0.876)
Yes	<18.5	4,336	705	23.8092	7.238(6.717,7.798)	6.3(5.846,6.79)
	18.5-24.9	214,817	10,698	6.8911	2.023(1.979,2.069)	1.936(1.892,1.98)
	≥25-	215,647	7,238	4.6115	1.436(1.4,1.474)	1.376(1.34,1.413)
≥ 65 years						
	Coronary heart disease					
No	No	689,427	90,193	18.4087	1.000(reference)	1.000(reference)
	Yes	62,266	10,764	24.8013	1.272(1.247,1.297)	1.316(1.289,1.342)
Yes	No	212,669	39,207	26.6458	1.492(1.475,1.51)	1.567(1.549,1.586)
	Yes	22,651	5,075	33.0544	1.797(1.747,1.849)	1.926(1.871,1.982)
	Ischemic stroke					
No	No	724,667	94,850	18.4153	1.000(reference)	1.000(reference)
	Yes	27,026	6,107	33.3942	1.606(1.565,1.648)	1.598(1.557,1.64)
Yes	No	225,328	41,372	26.5242	1.486(1.469,1.503)	1.557(1.538,1.575)
	Yes	9,992	2,910	44.655	2.325(2.241,2.412)	2.398(2.311,2.489)
	Estimated glomerular filtration rate (mL/min/1.73 m^2)					
No	>90	135,504	18,351	19.0552	1.000(reference)	1.000(reference)
	60-90	489,434	60,097	17.2174	0.921(0.905,0.936)	0.976(0.96,0.992)
	45-59	105,187	16,767	22.7382	1.138(1.115,1.163)	1.242(1.216,1.269)
	<45	21,568	5,742	40.2759	1.716(1.666,1.768)	1.866(1.811,1.923)
Yes	>90	37,458	6,686	25.7096	1.437(1.397,1.478)	1.56(1.517,1.604)

	60-90	142,440	23,769	23.9096	1.312(1.287,1.337)	1.484(1.456,1.514)
	45-59	43,056	9,220	31.365	1.638(1.598,1.68)	1.893(1.845,1.942)
	<45	12,366	4,607	59.9772	2.8(2.71,2.892)	3.245(3.14,3.353)
	Smoking					
No	No	660,142	81,164	17.2397	1.000(reference)	1.000(reference)
	Current	91,551	19,793	31.643	1.579(1.554,1.605)	1.444(1.42,1.468)
Yes	No	206,164	36,345	25.3759	1.511(1.492,1.53)	1.563(1.543,1.583)
	Current	29,156	7,937	41.1916	2.258(2.206,2.312)	2.18(2.129,2.232)
	Regular exercise					
No	No	471,920	70,666	21.2339	1.000(reference)	1.000(reference)
	Yes	279,773	30,291	15.1039	0.75(0.74,0.761)	0.777(0.766,0.788)
Yes	No	144,516	30,335	30.7038	1.502(1.482,1.522)	1.567(1.546,1.589)
	Yes	90,804	13,947	21.8962	1.107(1.087,1.127)	1.183(1.161,1.205)
	Abdominal obesity (Men:90cm/Women:85cm)					
No	No	406,979	64,588	22.6559	1.000(reference)	1.000(reference)
	Yes	344,714	36,369	14.6492	0.829(0.818,0.841)	1.151(1.133,1.169)
Yes	No	100,728	22,518	33.0716	1.505(1.482,1.528)	1.543(1.52,1.567)
	Yes	134,592	21,764	23.0536	1.28(1.26,1.301)	1.774(1.742,1.806)
	Body mass index (kg/m^2)					
No	<18.5	28,860	9,411	51.185	1.952(1.91,1.995)	1.892(1.851,1.934)
	18.5-25	461,252	66,683	20.4657	1.000(reference)	1.000(reference)
	25-	261,581	24,863	13.1457	0.781(0.77,0.793)	0.783(0.772,0.795)
Yes	<18.5	4,721	2,215	81.6377	3.288(3.151,3.43)	3.135(3.005,3.271)
	18.5-24.9	129,046	27,128	30.7961	1.551(1.529,1.573)	1.527(1.505,1.549)
	≥25-	101,553	14,939	20.8376	1.208(1.186,1.229)	1.183(1.161,1.204)

Supplementary Table S3. Incidence rate and MRR according to age. DM, diabetes mellitus; MRR, mortality rate ratio

Age (yr)	Total			Men			Women		
	No DM	DM	MRR	No DM	DM	MRR	No DM	DM	MRR
<40	0.048	0.120	2.517	0.057	0.129	2.271	0.029	0.069	2.413
40	0.081	0.246	3.037	0.099	0.261	2.636	0.061	0.208	3.410
41	0.093	0.266	2.860	0.113	0.304	2.690	0.049	0.080	1.633
42	0.093	0.239	2.570	0.117	0.282	2.410	0.065	0.129	1.985
43	0.101	0.239	2.366	0.125	0.272	2.176	0.056	0.100	1.786
44	0.112	0.284	2.536	0.151	0.347	2.298	0.069	0.135	1.957
45	0.118	0.283	2.398	0.152	0.313	2.059	0.061	0.164	2.689
46	0.128	0.307	2.398	0.176	0.377	2.142	0.079	0.145	1.835
47	0.152	0.319	2.099	0.192	0.377	1.964	0.087	0.103	1.184
48	0.145	0.355	2.448	0.212	0.439	2.071	0.084	0.188	2.238
49	0.170	0.381	2.241	0.220	0.449	2.041	0.097	0.159	1.639
50	0.175	0.414	2.366	0.259	0.519	2.004	0.104	0.220	2.115
51	0.204	0.483	2.368	0.270	0.557	2.063	0.109	0.252	2.312
52	0.206	0.475	2.306	0.314	0.611	1.946	0.119	0.245	2.059
53	0.245	0.557	2.273	0.331	0.667	2.015	0.124	0.245	1.976
54	0.235	0.523	2.226	0.373	0.708	1.898	0.135	0.255	1.889
55	0.287	0.641	2.233	0.392	0.789	2.013	0.143	0.266	1.860
56	0.280	0.611	2.182	0.452	0.867	1.918	0.153	0.275	1.797
57	0.361	0.673	1.864	0.496	0.821	1.655	0.181	0.334	1.845
58	0.346	0.696	2.012	0.554	0.964	1.740	0.189	0.373	1.974
59	0.424	0.889	2.097	0.617	1.116	1.809	0.184	0.432	2.348
60	0.398	0.738	1.854	0.640	1.055	1.648	0.215	0.371	1.726
61	0.519	0.879	1.694	0.722	1.141	1.580	0.266	0.400	1.504
62	0.481	0.923	1.919	0.749	1.310	1.749	0.274	0.509	1.858
63	0.599	1.004	1.676	0.851	1.268	1.490	0.290	0.561	1.934
64	0.622	1.035	1.664	0.959	1.429	1.490	0.357	0.632	1.770
65	0.757	1.321	1.745	1.074	1.752	1.631	0.402	0.705	1.754
66	0.805	1.392	1.729	1.251	1.934	1.546	0.469	0.886	1.889
67	1.086	1.814	1.670	1.486	2.268	1.526	0.591	1.135	1.920
68	0.991	1.709	1.725	1.522	2.433	1.599	0.583	1.048	1.798
69	1.284	2.076	1.617	1.837	2.739	1.491	0.763	1.329	1.742
70	1.321	2.081	1.575	2.035	2.973	1.461	0.771	1.303	1.690
71	1.632	2.524	1.547	2.370	3.504	1.478	0.987	1.612	1.633
72	1.700	2.568	1.511	2.575	3.640	1.414	1.034	1.687	1.632
73	2.140	3.127	1.461	3.171	4.365	1.377	1.355	2.180	1.609
74	2.174	3.281	1.509	3.238	4.594	1.419	1.388	2.263	1.630
75	2.706	3.813	1.409	4.150	5.494	1.324	1.789	2.732	1.527

76	2.839	3.972	1.399	4.176	5.471	1.310	1.889	2.958	1.566
77	3.492	4.878	1.397	5.226	6.749	1.291	2.537	3.842	1.514
78	3.573	4.847	1.357	5.135	6.643	1.294	2.546	3.780	1.485
79	4.361	5.884	1.349	6.373	8.249	1.294	3.417	4.838	1.416
80	4.597	6.132	1.334	6.173	7.793	1.262	3.531	5.080	1.439
81	5.648	7.152	1.266	7.459	9.803	1.314	4.829	5.943	1.231
82	5.834	7.361	1.262	7.737	9.347	1.208	4.591	6.224	1.356
83	7.549	8.738	1.158	10.514	12.006	1.142	6.253	7.599	1.215
84	7.584	9.870	1.301	9.616	12.609	1.311	6.215	8.267	1.330
85	9.025	9.840	1.090	12.172	12.329	1.013	7.456	8.849	1.187
86	8.835	10.612	1.201	10.503	12.169	1.159	7.647	9.613	1.257
87	10.453	14.855	1.421	12.306	16.348	1.328	9.671	14.114	1.459
88	10.674	13.012	1.219	11.730	17.053	1.454	9.909	10.562	1.066
89	11.234	14.787	1.316	14.048	21.290	1.516	10.068	12.372	1.229
90	12.538	13.586	1.084	12.756	14.861	1.165	12.361	12.693	1.027
≥90	16.012	19.063	1.191	17.614	21.191	1.203	15.271	18.032	1.181
